# Time to revisit arsenic regulations: comparing drinking water and rice

**DOI:** 10.1186/1471-2458-14-465

**Published:** 2014-05-17

**Authors:** Sébastien Sauvé

**Affiliations:** 1 Environmental Chemistry Laboratory, Chemistry Department, Université de Montréal, Montréal, QC, Canada

**Keywords:** Arsenic, Rice, Drinking water, Food safety, Cancer risks

## Abstract

**Background:**

Current arsenic regulations focus on drinking water without due consideration for dietary uptake and thus seem incoherent with respect to the risks arising from rice consumption. Existing arsenic guidelines are a cost-benefit compromise and, as such, they should be periodically re-evaluated.

**Discussion:**

Literature data was used to compare arsenic exposure from rice consumption relative to exposure arising from drinking water. Standard risk assessment paradigms show that arsenic regulations for drinking water should target a maximum concentration of nearly zero to prevent excessive lung and bladder cancer risks (among others). A *feasibility* threshold of 3 μg As l^-1^ was determined, but a cost-benefit analysis concluded that it would be too expensive to target a threshold below 10 μg As l^-1^. Data from the literature was used to compare exposure to arsenic from rice and rice product consumption relative to drinking water consumption. The exposure to arsenic from rice consumption can easily be equivalent to or greater than drinking water exposure that already exceeds standard risks and is based on feasibility and cost-benefit compromises. It must also be emphasized that many may disagree with the implications for their own health given the abnormally high cancer odds expected at the cost-benefit arsenic threshold.

**Summary:**

Tighter drinking water quality criteria should be implemented to properly protect people from excessive cancer risks. Food safety regulations must be put in place to prevent higher concentrations of arsenic in various drinks than those allowed in drinking water. Arsenic concentrations in rice should be regulated so as to roughly equate the risks and exposure levels observed from drinking water.

## Background

The toxicity of arsenic is well known: given its prominence in fiction and historical poisonings, it may be the most recognized toxic element in the periodic table. Nevertheless, much of the recognition and respect for arsenic’s toxicity seems to have been evacuated from water and food safety regulations. Arsenic is a trace element that occurs naturally in rocks and is present in soils and water. The widespread presence of traces of arsenic should therefore be expected. However, whether the arsenic in water and food originates from anthropogenic contamination or geochemical processes makes no difference to its toxicity. The chemical speciation of arsenic influences its toxicity and many organic forms of arsenic are presumed to have lower toxicity, such as in certain types of seafood, but most of the arsenic found in drinking water and rice is inorganic—the most toxic form.

Current water quality standards for most jurisdictions are 10 μg As l^-1^, a level presumed to adequately protect public health through a cost-benefit analysis. Even if the target should be zero, feasibility is estimated at 3 μg As l^-1^[1]. Surveys of various drinks yield surprising results: many rice milk drinks and fruit juices have arsenic concentrations that are up to six times higher than what is deemed safe in water (from 6 to 59 with a mean of 23 μg As l^-1^ in rice drinks [2,3]). Given that some children drink up to half a litre of fruit juice per day [3] and that lactose-intolerant people may consume significant amounts of rice milk drinks, the resulting dose of arsenic would be higher than the level considered safe. If a threshold for As in water is deemed relatively safe at 10 μg l^-1^ for people drinking up two 2 litres of water per day, how can up to 60 μg As l^-1^ be safe for people drinking nearly equivalent amounts of juice or rice drinks?

Where does the arsenic in fruit juices come from? In North America, DDT began to replace arsenic pesticides in the early 1950s. Though arsenic was gradually phased out and banned across the continent in the 1980s [4], many orchards and fields are still contaminated. The higher concentrations of arsenic in fruit juices are presumed to arise from the arsenic remaining in the soils of orchards where the As-pesticides were spread. The residual arsenic from the pesticides in the soil is taken up by the plant roots and partly transferred to the fruit.

**Figure 1:**
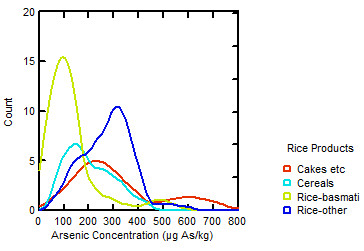
**Density function distribution of arsenic concentrations in the 165 rice products analysed in the USFDA survey **[2]**.**

And rice? Rice has, by far, a much higher As content than any other plant food product grown under normal conditions [5,6]. Only fish and seafood may post higher concentrations. Some of the higher As content in rice may also be attributable to residual pesticides in the soils. Certain data suggest that genetic selection could have inadvertently aggravated the situation by promoting traits for As accumulation [6,7]. Nevertheless, most of the arsenic accumulation probably arises from the particularity of the rice plant and its culture. Arsenic is a rather soluble trace element and has a much higher propensity to partition into the soil interstitial water relative to soil solids as compared to most other trace elements. Rice is usually grown in flooded or very wet conditions, optimizing the transfer of As from the soil solids to the interstitial soil solution, which is then taken up into the roots of the plants and transferred to the grains. When combined with genetic selection that could promote the accumulation of As in rice and irrigation water that may already contain significant levels of arsenic, higher concentrations of arsenic tend to occur in rice more than in any other cereal. An analysis of 900 rice grains from various origins shows a very wide range of concentrations with mean As concentrations by country varying from the low range in Egypt and India (50–70) to the high range in the USA and France (250–280 μg As kg^-1^ of rice dry weight [8]), suggesting that the combination of environmental conditions and rice variety has a significant impact on arsenic accumulation and that low arsenic levels are possible. Figure 1 illustrates the range of arsenic concentrations reported in 165 rice and rice-based products analysed by the USFDA [2]. Non-basmati rice and the rice cakes and crackers group contain significantly higher concentrations of arsenic than basmati rice or rice-based cereals (Tukey’s post hoc ANOVA p < 0.05). It should be noted that the lower levels found in basmati rice may be explained, at least in part, by the fact that the grain is often imported from countries with lower arsenic concentrations in rice.

## Discussion

The concentration of arsenic in rice is subject to a standard threshold regulation of 1 mg As kg^-1^ (1000 μg As kg^-1^ if we use the same units). This level is inherited from previous risk assessments across a wide range of food products. However, several recent studies on the levels of arsenic in rice and rice-based products have provided sufficient evidence and data to question this overly generous threshold, specifically with regards to rice. Currently, there are no effective regulations for arsenic concentrations in rice set by the WHO, EU or US agencies [6]. China has the strictest regulations for arsenic in rice at 150 μg As_inorganic_ kg^-1^, but, since rice is such a staple food in Asia, rice consumption remains a higher source of exposure than drinking water.

Given the lack of effective regulations in most developed countries, highly As-contaminated rice may be sold and unknowingly consumed. The USFDA study analysed arsenic concentrations in 165 different rice products, yielding a mean of 222 ± 135 μg As kg^-1^ with a range of concentrations from <4 μg As kg^-1^ to 723 μg As kg^-1^ - [2]. Using the mean concentration of As in the survey, it is possible to estimate that eating 90 g of rice (dry weight before cooking – roughly equivalent to a normal serving of 250–300 g of cooked rice) will deliver the same amount of arsenic as drinking 2 litres of water at 10 μg As l^-1^. An upper-bound estimate of exposure (based on the maximum concentration rather than the mean) indicates that 28 g of rice (dry weight before cooking) provides the same As intake as 2 litres of water at 10 μg As l^-1^. The As concentrations in rice reported by the USFDA are consistent with available literature data and not exceptional, so, within the variability expected from such agricultural crops, these estimates are representative of arsenic exposure through rice consumption in the USA (rice from other countries sometimes has lower arsenic levels - [6,8]).

Considering that rice can be an important source of As, it seems necessary and urgent that a meaningful As regulations be established for this staple food. Moderate rice consumption has been clearly shown to increase urinary excretion of arsenic in children from the general US population [5]. A study of 229 pregnant women showed that both rice consumption and arsenic exposure through water were associated with significantly higher levels of arsenic in urine [9]. Another study in Bangladesh showed significant correlations between rice consumption, urinary arsenic and skin lesions associated with arsenic exposure [10]. The gastrointestinal bioavailability of arsenic from rice should certainly be considered, though most studies demonstrate that increased rice consumption leads to increased arsenic exposure and greater risks.

The literature clearly demonstrates that the bioavailability of arsenic is highly dependent on its chemical speciation and growth conditions. For example, there are significant differences in using greenhouse-grown rice under test conditions versus store-bought rice [11]. For rice grown in test conditions conducive to the presence of organic forms of arsenic, the bioavailability was only about 33%, whereas the store-bought standard rice laden with inorganic arsenic had a bioavailability of 89% [11]. In a large study (901 rice samples), Meharg et al. looked at variations in the levels of inorganic arsenic in rice from various origins and found that the trends of As speciation in rice vary according to where the rice was grown [8]. Tests on the gastrointestinal bioaccessibility of arsenic show that As availability remains relatively high at 63 to 99% [12]. In most studies, a high proportion of total rice arsenic occurs as inorganic arsenic (80 to 91% [13]). In sum, arsenic in rice usually occurs mainly as inorganic arsenic, which has a high bioavailability.

Arsenic has been linked to several adverse health outcomes, including lung cancer and lung disease [14], liver cancer [15], cardiovascular disease [16], possibly diabetes [17] and other ailments (see [15] for an extensive table of published results). The aforementioned rice exposure calculations are based on the assumption that the current guideline of 10 μg As l^-1^ in drinking water is safe (equivalent to a reference dose of 0.3 μg kg^-1^ d^-1^[18]). This level roughly corresponds to what current risk assessment procedure and toxicology would recognize as safe to protect against the toxic effects caused by excessive exposure to arsenic. But the main concern for arsenic is not so much acute immediate toxicity; it is rather its carcinogenic properties and the long-term implications. The usual level of acceptable risk for carcinogens is 10^-6^ (i.e. a Russian roulette of 1 in a million chance of getting cancer over a lifetime). In some cases, such as professional exposure where workers are aware of the risks, we usually accept less favourable odds. Standards also depend on technical feasibility to reduce exposure level. Because groundwater may naturally contain relatively high concentrations of geochemical arsenic, the drinking water quality guidelines are a *cost-benefit* compromise to prevent the excessive costs associated with labelling a large number of private wells and groundwater sources as contaminated. Nevertheless, the technological means to remove arsenic from drinking water in developed countries exist and are neither outrageously expensive nor complex. This *technological feasibility* compromise has been estimated at 3 μg As l^-1^, and the decision to use a 10 μg As l^-1^ threshold instead of 3 is therefore mainly a budgetary decision [1]. It should be emphasized that the choice is more motivated by politics than by technology (i.e. authorities do not want to stigmatize geographical regions where groundwater used for human consumption is contaminated with arsenic and thus impose the economic burden of water treatment on the local populations). As a result, many people drink water at levels very close to the current guideline of 10 μg As l^-1^, and, even if they consciously have their well water analysed, they may not be aware that they are exposed to an increased risk of cancer (using recognized risk assessment standards). Even worse, many groundwater sources currently distribute water much above 10 μg As l^-1^, and the people living in these regions must understand that current arsenic guidelines are only marginally protective. Awareness programs should therefore be implemented or enhanced wherever needed. It would also be ethical to ensure that people are aware that current arsenic regulations are a cost-benefit compromise and that, based on usual health risk paradigms, the standards should be much lower.

It is certainly difficult to precisely evaluate the excess lifetime risks of cancer associated with arsenic exposure, partly because of the already elevated background risks associated with such cancers types (mainly bladder and lung) and because very large study populations are required for proper statistical analysis [19]. It is also very difficult to dissociate As exposure from drinking water consumption from exposure from food. In fact, the issue is possibly aggravated in regions where rice is grown locally and the health effects of arsenic are the result of overall exposure from contaminated water and food-borne contaminants. The difficulties in distinguishing exposure from drinking water and food and the few large-scale epidemiological studies available may help explain why the link between cancer and arsenic-laden rice has not been clearly demonstrated. Research efforts must focus on a better understanding of the contribution of rice consumption to lifetime excess cancer risks.

For the most part, arsenic risks are calculated from exposure events at relatively high As concentrations and extrapolated to lower levels. Certain assumptions and much uncertainty are involved in this process. Based on current data, the reference maximum daily dose for cancer risk from arsenic is estimated at between 3.7 · 10^-7^ mg/kg and 6.7 · 10^-7^ mg/kg [20-22], which would translate into a drinking water quality guideline of 0.02 μg As l^-1^ for an adult drinking two litres per day (6.7 · 10^-4^ μg/kg * 70 kg/2 liters) if we were to use the normally accepted 10^-6^ odds of cancer risk [22]. While this is a rather drastic change from the threshold of 10 μg As l^-1^, it underlines just how little precaution is instilled in the current guidelines. The same approach suggests that a drinking water regulation of 3 μg As l^-1^ would ensure a level of protection that is roughly equivalent to 1 in 10 000 (10^-4^ risks) [22]. There are many uncertainties with this estimate, and it is therefore too radical to expect a sudden and steep change in the guidelines to the standard 10^-6^ cancer risk level, which would be nearly impossible to implement at 0.02 μg As l^-1^. Nevertheless, the current regulations for arsenic are roughly 500 times higher than those that would normally be targeted for protection against cancer risks and seem to defy generally accepted risk levels [23]. Though such a sharp deviation from normal procedures was proposed as a compromise given the prevalence of arsenic, it certainly warrants periodic reassessments. Also, this initial risk assessment did not consider the added exposure from rice consumption.

The calculations of a cost-benefit threshold relative to one’s cancer risk is an intimately personal decision, and people must be aware that regulatory targets for arsenic should be as close to zero as possible and that the technological means to aim for 3 μg As l^-1^ are within reach.

A critical challenge to defining a safe level of exposure to arsenic is to establish how the obvious causal cancer link due to high arsenic exposure, which is widely accepted, can be extrapolated to what we can expect from lower exposure [24], which is much debated. Humans may tolerate a safe low-level exposure to arsenic, but this is counter-current to the usual cancer risk paradigm and has yet to be demonstrated. Still, exposing mice to an As dose equivalent to the current guideline has been shown to demonstrate genotoxicity [18]—another clue that the threshold must be revisited. An initial guideline target of 3 μg l^-1^ could be set for future implementation, thus allowing municipalities to address the issue so that their aqueducts are not suddenly declared improper for consumption. Setting this type of threshold would also certainly increase overall awareness of the problem and generate significant public pressure to correct the situation where needed. Individuals with private wells above the threshold could install an appropriate water treatment device to ensure that their water is safer.

Current data suggest that the water quality guidelines should be systematically enforced for all beverages, without exception, there does not seem to be a sound scientific basis to allow higher As concentrations in drinks consumed in quantities equivalent to water. As for rice, it is possible to produce crops that contain relatively low levels of arsenic, as shown in the USFDA survey of rice products (Figure 1) and others [2,8]. One may presume that if the right soil, agricultural practices and rice varieties are used, the rice will contain much lower levels of arsenic [25,26]. It is possible to compare available toxicological data for drinking water and the risks from rice consumption. Based on the proposed 3 μg l^-1^ water quality threshold and an equivalent exposure from eating 100 g of rice (dry weight before cooking – an average daily portion of about 250–300 g of cooked rice – two culinary cups), a guideline for rice at 60 μg As kg^-1^ emerges. While it would be a challenge for some rice producers, the threshold would ensure some protection against excessive cancer risks. The rice producer lobby may say that it is impossible to remain under the threshold and seek to continue to sell the same As-laden rice, but it is very likely that, should the regulations be implemented, rice varieties with low As absorption would be very quickly identified and the most As-rich production areas would be switched to other types of agricultural crops that do not accumulate arsenic. While the chemical speciation of arsenic in rice is important, available data seem to suggest that roughly one- to two-thirds of total arsenic in rice occurs as inorganic arsenic [2,6,27,28], which has been identified as the main concern. Furthermore, even if we were to agree that the current 10 μg As l^-1^ drinking water threshold is safe, rice concentration would still need to be limited to <200 μg As kg^-1^ (see Figure 1) in order to ensure that eating 100 g of rice (dry weight before cooking) does not cause an equivalent exposure. This level would be roughly equivalent to what is used for drinking water, presuming a moderate daily consumption of rice. Then again, if the water consumed is already at the threshold, eating 100 g of As rice effectively doubles the exposure to arsenic, and current guidelines presume that drinking water is the main source of exposure. An intermediate approach could be to propose a standard for rice at 100 μg As kg^-1^ to match a 3 μg As l^-1^ water quality standard, presuming that some of the As in rice occurs in an organic form and that the bioavailability of As is probably lower in rice than in drinking water. This level would still represent a significant implementation challenge but, given that it is the mean value for 110 Egyptian rice samples [8], it is certainly not impossible. It is of interest to note that these levels (60 to 200 μg As kg^-1^) are lower but in the same order of magnitude as the maximum level of 300 μg As kg^-1^ proposed for discussion by FAO/WHO [29].

## Summary

The scientific basis that allows for a roughly 500 times higher risk of cancer from arsenic in drinking water must be questioned as compared to the standard toxicological and cancer risk assessment paradigms used for most other potential contaminants (another such exception is uranium). There is much uncertainty as to how we should extrapolate the obvious cancer risks observed for high As concentrations in drinking water to what would be safe cancer risk levels at much lower concentrations. While scientists and lobby groups debate the issue, those who consume groundwater should have it tested for arsenic and decide for themselves what sort of precautionary/cost-benefit approach they want to take with regards to their own health. We should also curtail or strongly limit our consumption of rice while authorities ponder when and how they will regulate arsenic concentration in rice. To be coherent with current drinking water quality regulations, an initial arsenic threshold for rice must be urgently implemented so that arsenic exposure from rice consumption is roughly equivalent to what we accept for drinking water. This initial level should be around 200 μg As kg^-1^ so as to be coherent with the current 10 μg As l^-1^ drinking water threshold. A further tightening of arsenic regulations to 3 μg As l^-1^ in drinking water and eventually 100 μg As kg^-1^ in rice would still represent significant and above-normal cancer risks but would better reflect the *technological* and *cost-benefit* limitations of what risk management can really achieve and reduce potential adverse health effects inasmuch as possible.

## Competing interests

The author declares that he has no competing interests.

## Pre-publication history

The pre-publication history for this paper can be accessed here:

http://www.biomedcentral.com/1471-2458/14/465/prepub
